# Western medical ethics taught to junior medical students can cross cultural and linguistic boundaries

**DOI:** 10.1186/1472-6939-5-4

**Published:** 2004-07-30

**Authors:** Valmae A Ypinazar, Stephen A Margolis

**Affiliations:** 1 School of Education, James Cook University, Townsville, Queensland, 4810, Australia; 2 Department of General Practice, Monash University, 867 Centre Road, East Bentleigh, Victoria, 3165, Australia

## Abstract

**Background:**

Little is known about teaching medical ethics across cultural and linguistic boundaries. This study examined two successive cohorts of first year medical students in a six year undergraduate MBBS program.

**Methods:**

The objective was to investigate whether Arabic speaking students studying medicine in an Arabic country would be able to correctly identify some of the principles of Western medical ethical reasoning. This cohort study was conducted on first year students in a six-year undergraduate program studying medicine in English, their second language at a medical school in the Arabian Gulf. The ethics teaching was based on the four-principle approach (autonomy, beneficence, non-malfeasance and justice) and delivered by a non-Muslim native English speaker with no knowledge of the Arabic language. Although the course was respectful of Arabic culture and tradition, the content excluded an analysis of Islamic medical ethics and focused on Western ethical reasoning. Following two 45-minute interactive seminars, students in groups of 3 or 4 visited a primary health care centre for one morning, sitting in with an attending physician seeing his or her patients in Arabic. Each student submitted a personal report for summative assessment detailing the ethical issues they had observed.

**Results:**

All 62 students enrolled in these courses participated. Each student acting independently was able to correctly identify a median number of 4 different medical ethical issues (range 2–9) and correctly identify and label accurately a median of 2 different medical ethical issues (range 2–7) There were no significant correlations between their English language skills or general academic ability and the number or accuracy of ethical issues identified.

**Conclusions:**

This study has demonstrated that these students could identify medical ethical issues based on Western constructs, despite learning in English, their second language, being in the third week of their medical school experience and with minimal instruction. This result was independent of their academic and English language skills suggesting that ethical principles as espoused in the four principal approach may be common to the students' Islamic religious beliefs, allowing them to access complex medical ethical reasoning skills at an early stage in the medical curriculum.

## Background

Medical ethics has increasingly become a common component of the undergraduate curriculum at many medical schools, often within a defined humanities program [1-3]. In 1999, the European Federation of Internal Medicine, the American College of Physicians and the American Board of Internal Medicine launched the Medical Professionalism Project, which placed medical ethics at the centre of a charter of behaviour and attitudes for all physicians [4]. In 2003, the Liaison Committee on Medical Education in the USA identified the teaching of medical ethics as a core curriculum component of modern medical school education [5].

Medical ethics teaching can occur within the traditional model of medical education, where a separate program of clinical instruction follows several years of medical sciences [6]. However, there is some evidence to suggest that a vertically integrated learning model where the study of clinical subjects runs parallels to and is integrated with basic sciences may be more effective [7,8]. This has resulted in a trend towards including clinical experience in the early years of the medical school curriculum. There is increasing evidence that this enhances student attitudes towards patients [9], provides a structure for teaching integrated clinical medicine [10] and better prepares students for the clinical years [11].

Although Islam has a long tradition of ethical reasoning, there had been little impetus to establish courses in ethics within medical school curricula in the Arabian Gulf. The Faculty of Medicine and Health Sciences (FMHS) was almost alone in having done so, an initiative introduced by expatriate family physicians from New Zealand who had undertaken training in Western medical ethics. Ethics had been taught at the FMHS since 1997 in a vertically integrated program that spanned all levels of the curriculum, based on the four-principle approach developed by Beauchamp and Childress [12,13] as modified by Herbert; autonomy, beneficence, non-malfeasance and justice [14,15] (See Table 1). Although it was less than ideal to focus the medical ethics curriculum on an imported model, there was little support within the UAE University for a course centred on local culture and traditions.

**Table 1:** Ethical Principles utilized in the teaching program

*Primary Principle*	*Subsection principles*
1. Autonomy	
	a) Disclosure
	b) Truth telling
	c) Informed consent
	d) Competence
	e) Paternalism
	f) Confidentiality
	g) Decision-making
2. Beneficence	
3. Non-malfeasance	
4. Justice	

English has become the lingua franca of medicine, with most international medical societies, publications and meetings conducted in English [16,17]. This has encouraged the development of English language medical schools in countries where English is a second language, including the Arabian Gulf.

There is a significant body of literature describing how people from different cultures utilise different approaches to the clinical application of medical ethics [18,19]. However, there is little information about teaching medical ethics across cultural and linguistic boundaries. Social anthropology has demonstrated that there is a strategic mixing of language with culture and that both culture and social systems are conceptualised in language [20,21]. This would suggest that students learning medicine in English, their second language, could experience dissonance between the content of their courses and the context of their everyday lives, especially in socially formulated issues such as medical ethics.

The aim of this study was to investigate whether Arabic speaking students studying medicine in an Arabic country, albeit with the language of instruction being English, would be able to correctly identify some of the principles of Western medical ethical reasoning, a discipline that challenged significant cultural and linguistic boundaries.

## Methods

### Setting

The FMHS at the United Arab Emirates (UAE) University offers a six-year undergraduate program in medicine. Students are admitted after a one-year post high school tertiary orientation year, which includes additional English language instruction. Although all students are citizens of the UAE, speaking Arabic as their first language, the course is conducted in English therefore students are required to meet an English language proficiency level in order to be accepted into the medical school. Faculty members come from across the globe, with approximately 1/3 with English as their first language, 1/3 with Arabic as their first language and 1/3 with a different primary language.

All medical students participated in an introductory course in medical ethics, conducted in the first three weeks of first year and taught by a non-Muslim native English speaker from New Zealand with no knowledge of the Arabic language. Apart from his qualifications in Family Medicine, he had also undergone training in Western medical ethics. Although the course was respectful of Arabic culture and tradition, the content of the introductory course focused on Western medical ethical reasoning and excluded an analysis of Islamic medical ethics.

Based on the principles of adult learning, the educational strategy focused on the use of real clinical encounters after minimal orientation, followed by an interactive debriefing session. Rather than provide a sound basis for ethical reasoning, the aim of the introductory course was to introduce the students to the central importance of ethical principles in medical practice, knowing that a more complete and integrated understanding would develop over the ensuing six years of the course. Following two 45-minute interactive seminars, students in groups of 3 or 4 visited a primary health care centre for one morning, sitting in with an attending physician seeing his or her patients. In the absence of any clinical understanding at this early point in their training, the students were encouraged to focus all their energies on medical ethics.

Following the visit to the primary health care centre each student submitted a personal report for summative assessment in which they described the ethical situations they had observed and then provided an appropriate label according to the four-principal approach. Although the seminars were in held in English, the clinical sessions were conducted in Arabic. When questioned by the investigator (SM), the clinical staff stated they had never received formal instruction in medical ethics. The clinical staff were discouraged from indicating ethical issues to the students; the educational process was for the students to identify these on their own. Following submission of their personal report, a third seminar of the introductory course was held with the same teacher where students were invited to share and discuss their experiences.

### Sample

This study examined two cohorts of first year students, 33 students from the 2002 – 2003 academic year and 29 students from the 2003 – 2004 academic year (i.e. all students enrolled in these courses). Each cohort experienced the same admission criteria, underwent the same curriculum, was taught ethics by the same native English-speaking teacher and utilised the same group of primary health care physicians.

### Analysis methodology

Although both experienced educators and educational researchers, the investigators (VY and SM) did not teach medical ethics at this level of the curriculum. Each investigator coded all personal reports independently and any discrepancies were resolved by mutual agreement. A positive score for labelling any of the four principles and the subsets of autonomy was only allocated when the accompanying description corresponded with the label given. Merely listing a label was not coded as positive.

As an indicator of general academic ability, the students' score on a multidisciplinary unit test held at the end of the fifth week of year one was also considered. The results of this test have been considered as a variable in the study to determine if there was a correlation between general academic ability and the students' competency in identifying ethical principals and to avoid associated bias. This multidisciplinary unit test covered all topics taught in the first 5 weeks including anatomy, chemistry, medical physics and medical ethics. The pass mark was set at 75%. The medical ethics component contributed 5% of the total mark.

As not all students had the same clinical experience, each individual student may not have had the opportunity to observe the same range of ethical issues during their clinical experience. For example, some students may not have seen an example of competence to detail in their report. Hence, as this manuscript concerns each individual student's ability to report what he or she as an individual had observed in an experience not shared across the whole group, only numbers of ethical issues identified by individual students are reported rather than the total numbers of students per ethical issue.

A third variable included was their score in a standardised test of English, the Test of English as a Foreign Language (TOEFL) [22], used in the selection process to enter medical school. Although initially designed as a measure of the English language proficiency of international students wishing to study at colleges and universities in the North America, this test was widely used in the Arabian Gulf. The TOEFL is a multiple-choice examination that measured listening, comprehension, vocabulary and reading [23]. This test measures the English language proficiency of non-native speakers of English and as such has relevance in the FMHS as the medical course there is taught in English. The nominal requirement at the FMHS is a minimum score of 500. As this test has a non-linear scoring system, this is substantially lower than the 550 – 580 required in Western universities in North America, Australasia and the UK.

The Statistical Package for the Social Sciences was used for analysing the results [24]. Simple frequency analysis was used to describe demographics, TOEFL scores, unit test results and quantification of ethics issues. The correlation between the TOEFL score and Multidisciplinary Unit Test Score was assessed by Pearson's correlation coefficient as both variables were normally distributed and held a linear relationship. As the number of ethical issues identified was not distributed in a Gaussian fashion, frequency distributions were reported by median and range from minimum to maximum, while the appropriate non-parametric statistics were used: Mann-Whitney U test for comparisons between variables and Kendall's tau-b test for bivariate correlations. The level of statistical significance was defined as p < 0.05.

The Research Ethics Committee of the United Arab Emirates University Faculty of Medicine and Health Sciences, which complies with the ethical rules for human experimentation that are stated in the Declaration of Helsinki, approved the project.

## Results

All members of each class participated in this study. Participant demographics are detailed in Table 2. The age range was from 19–21 years; all were practicing Muslims and had Arabic as their first language.

**Table 2:** Participant demographics, TOEFL score and test results

	Class			
	Cohort 2002–2003		Cohort 2003–2004	

	Male	Female	Male	Female

n	13	20	11	18

TOEFL* score prior to entry to medical school	498 +/- 27.8	518 +/- 58.2	514.9 +/- 40.9	505.6 +/- 29.0
Multidisciplinary Unit Test Score [pass = 75%]	82.8 +/- 5.1	82.7 +/- 6.6	81.9 +/- 6.8	83.9 +/- 3.6

[All enrolled students in this course participated] * The Test of English as a Foreign Language

The similarities of issues identified and described across the group of students who attended the same clinic were highly suggestive that the events described actually occurred. However, there were sufficient differences in identified principles, evidence provided and labels given, to indicate that reports were written independently.

Table 3 details the ethical issues identified. Although the initial expectation was that there were only a limited number of ethical issues that could be identified in a single clinical session, each student acting independently was able to correctly identify a median number of 4 different medical ethical issues (range 2–9) and correctly identify and label accurately a median of 2 different medical ethical issues (range 2–7) There was no significant difference in results between males and females except for 'autonomy mislabelled as a different ethical issue' (male: median = 0, range= 0–2; female median = 0, range = 0–3; p = 0.002) and 'autonomy / confidentiality mislabelled as a different ethical issue' (male: all results = 0; female median = 0, range = 0 – 2; p = 0.03).

**Table 3:** Analysis of Ethics assignments: the median and range from minimum to maximum of the number of issues identified by individual students as detailed in their personal report

	Median	Range
Total number of ethical issues identified:		
Labelled correctly	2	2 – 7
Mislabelled as a different ethical issue or subset of Autonomy not defined	2	3 – 9
[Labelled correctly] + [mislabelled]	4	2 – 9
Total number of issues identified as ethical which were not ethical issues	0	0–4
Total number of autonomy issues identified		
Labelled correctly	1	0–4
Labelled as Autonomy, no subsection specified	0	0–3
Mis-labelled as a different ethical issue	0	0–3
Total number of autonomy: disclosure issues identified		
Labelled correctly	0	0–1
Labelled as Autonomy, no subsection specified	0	0–1
Mis-labelled as a different ethical issue	0	0–1
Total number of autonomy: truth telling issues identified		
Labelled correctly	0	0–1
Labelled as Autonomy, no subsection specified	0	0–1
Mis-labelled as a different ethical issue	0	0 – 0
Total number of autonomy: informed consent issues identified		
Labelled correctly	0	0 – 2
Labelled as Autonomy, no subsection specified	0	0 – 1
Mis-labelled as a different ethical issue	0	0 – 1
Total number of autonomy: competence issues identified		
Labelled correctly	0	0 – 0
Labelled as Autonomy, no subsection specified	0	0 – 1
Mis-labelled as a different ethical issue	0	0 – 0
Total number of autonomy: paternalism issues identified		
Labelled correctly	0	0 – 1
Labelled as Autonomy, no subsection specified	0	0 – 1
Mis-labelled as a different ethical issue	0	0 – 1
Total number of autonomy: confidentiality issues identified		
Labelled correctly	0	0 – 1
Labelled as Autonomy, no subsection specified	0	0 – 1
Mis-labelled as a different ethical issue	0	0 – 2
Total number of autonomy: decision – making issues identified		
Labelled correctly	0	0 – 2
Labelled as Autonomy, no subsection specified	0	0 – 2
Mis-labelled as a different ethical issue	0	0 – 2
Total number of beneficence issues identified		
Labelled correctly	0	0 – 2
Mis-labelled as a different ethical issue	0	0 – 1
Total number of non-malfeasance issues identified		
Labelled correctly	0	0 – 2
Mis-labelled as a different ethical issue	0	0 – 3
Total number of justice issues identified		
Labelled correctly	0	0 – 1
Mis-labelled as a different ethical issue	0	0 – 2

The four cohorts of students displayed a wide range of TOEFL scores ranging from the low 400 s through the mid 600 s. There was a moderate correlation between the TOEFL score and the multidisciplinary unit test score (r = 0.616, p < 0.001). There was no statistically significant correlation between the TOEFL score and the number of ethical issues identified. There were weak statistically significant correlations between the multidisciplinary unit test scores and ethical issues identified for 'total number of issues correctly identified' (r = 0.26, p = 0.008) and 'number of autonomy issues correctly identified' (r = 0.21, p = 0.04). There were no other statistically significant correlations between test scores and ethical issues identified.

## Discussion

This study has demonstrated that these first year students were able to identify medical ethical issues in a clinical setting after minimal instruction. Although introductory courses by their very nature aim to build on the students' previously accumulated knowledge, understanding and experience, especially the unstructured kind based on life experience, the students in this study were able to develop this skill with very minimal instruction, when compared to the normal length of introductory medical ethics courses [25].

Feldman et al found important differences in the ethical practices and beliefs of Internists in the USA and China, even amongst those practicing Western medicine and suggested that some basic bioethical principles may be culturally based rather than universal [26]. One important feature in this regard is the concept of autonomy, where more traditional or tribal based societies, such as is seen in the Arabian Gulf States, often view autonomy as relating to the group rather than the individual. Hence, students learning ethics that incorporate Western constructs and are delivered in a Western language may fail to see the relevance or appropriateness to their understanding of their culture and society. However, this study has demonstrated that the students were able to correctly identify the concept of autonomy and some of its sub groupings.

Although the students in this study demonstrated that they were able to use an imported model of ethical principles, this study did not directly address whether the students embraced the underlying conceptual framework, as the educational objectives of the introductory course were primarily to arouse interest and discussion amongst the students. However, informal review of the performance in examination settings of earlier cohorts of students who underwent similar educational programs suggests that by the end of their pre-registration medical training students do internalise the concepts and appear to understand their clinical applications. Further studies could investigate the impact of ethical training on their eventual behaviour as independent clinicians.

This study had an underlying a priori assumption that the students would not be successful in identifying ethical issues due to a combination of the brief nature of the introductory seminars, their relative youth and inexperience with the adult learning model and being at the beginning of their medical training. Hence, the relatively high number of issues identified and the acceptable accuracy in labelling suggests that these students did demonstrate a reasonable level of skill even in the absence of a control group to help validate this conclusion. With little research available on the impact of early education in medical ethics, further studies utilising a control group would prove beneficial to determine what issues students would have been able to identify with no training.

With ethics and professionalism being globally recognised as an integral component of primary medical degree curriculum, the results of the project suggest that early exposure may be beneficial in that it would provide a solid base in ethics, an essential requirement for their later clinical training. If ethical principles are clearly established early in the students' training, all future clinical training can be considered from an ethical standpoint.

Jackson found that corporate managers' ethical decision-making was more influenced by their home country than by the country in which they resided [27]. This suggests the overriding importance culture and tradition has on one's values and beliefs systems. Hence the absence of a correlation between English language ability, accuracy in identification of ethical issues and number of issues identified suggests that in this case the basic conceptualization of medical ethics transcended the possible values and beliefs systems implicit in teaching a Western ethical curriculum in the English language in this environment.

A number of researchers have suggested that the four principles of Western medical ethics have always existed in Islam [28-30]. Islam has an ethical and moral tradition which is intimately linked to Qur'anic teachings [31]. While the students would be familiar with ethical principles as enshrined in their religious beliefs, the linguistic constructs and labels of Western medical ethics as outlined in the four principle approach would be unfamiliar to these students. Perhaps the ethical principles embedded in their religion enabled the students to juxtapose Western linguistic constructs onto the ethical events observed in the clinical situation. They demonstrated an ability to correctly identify, describe and label ethical events despite using new language constructs, given that English is their second language. The relative importance of the intervention compared to their background knowledge could be further addressed in an expanded study using a control group.

This study found at best a weak correlation between general academic ability and the number and accuracy of ethics issues identified. This suggests that even academically weak students were able to grasp the concepts being taught. Perhaps this outcome was enhanced by two factors; utilizing an adult learning model where students worked together in groups (although assignments were individually prepared) undergoing real rather than simulated experiences and the incorporation of their Islamic religious beliefs and self developed ethical reasoning.

The students in this study had a broad range of English language skills as suggested by the large range in TOEFL results. Although there is no clear evidence that TOEFL correlates with the ability to conceptualise theoretical constructs presented in English, there is strong evidence that TOEFL correlates with grade point average at a North American Universities [32,33], and some evidence it correlates with the degree of participation of UAE students in problem based learning sessions [34].

This study only addressed the ability of medical students at the onset of their medical training to assimilate and conceptualise the basic principles of ethical reasoning. In particular, no assessment was made of the long-term impact of this course on 'moral enculturation' as this study has only described one component of a longitudinal six-year course in medical ethics.

## Conclusions

This study has demonstrated that these students could identify medical ethical issues based on Western constructs, despite learning in English, their second language, being in the third week of their medical school experience and with minimal instruction. This result was independent of their academic and English language skills suggesting that ethical principles as espoused in the four principal approach may be common to the students' Islamic religious beliefs, allowing them to access complex medical ethical reasoning skills at an early stage in the medical curriculum.

## Competing interests

None declared.

## Authors' contributions

SM conceived the study, prepared the ethics application, coded the student's material, analysed the results, prepared and reviewed the manuscript. VY prepared the ethics application, prepared the coding sheet, coded the student's material, analysed the results, prepared and reviewed the manuscript.

## Pre-publication history

The pre-publication history for this paper can be accessed here:



## References

[B1] Murray J (2003). Development of a medical humanities program at Dalhousie University Faculty of Medicine, Nova Scotia, Canada, 1992-2003. Acad Med.

[B2] Montgomery K, Chambers T, Reifler DR (2003). Humanities Education at Northwestern University's Feinberg School of Medicine. Acad Med.

[B3] Andre J, Brody H, Fleck L, Thomason CL, Tomlinson T (2003). Ethics, professionalism, and humanities at Michigan State University College of Human Medicine. Acad Med.

[B4] (2002). Medical Professionalism in the new millennium: a physician charter. Ann Intern Med.

[B5] LCME Secretariat (2003). Functions and Structure of a Medical School: Standards for Accreditation of Medical Education Programs Leading to the MD Degree.

[B6] Flexner A (1910). Medical education in the United States and Canada. Carnegie Foundation for the Advancement of Teaching.

[B7] Harden R,M (1998). Integrated Teaching - what do we mean? A proposed taxonomy.. Medical Education.

[B8] Dahle L,O, Brynhildsen J, Berbohm Fallsberg M, Rundquist I, Hammar M (2002). Pros and cons of vertical integration between clinical medicine and basic science within a problem-based undergraduate medical curriculum: examples and experience from Linkoping, Sweden.. Medical Teacher.

[B9] Chisholm Marie A., Wade William E. (1999). Using Actual Patients in the Classroom To Develop Positive Student Attitudes Toward Pharmaceutical Care. American Journal of Pharmaceutical Education.

[B10] Matson CC, Ullian JA, Boisaubin EV (1999). Intergrating early clinical experience curricula at two medical schools: Lessons learned from the Robert Wood Johnson Foundation's Generalist Physician Initiative. Academic Medicine.

[B11] Lofaro M J, Abernathy C M (1994). An innovative course in surgical critical care for second year medical students. Academic Medicine.

[B12] Gillon R, Beauchamp TL (1994). The four principles' approach. Principles of health care ethics.

[B13] Beauchamp TL, Childress J (1989). Principles of biomedical ethics.

[B14] Herbert PC (1996). Doing right: a practical guide to ethics for medical trainees and physicians..

[B15] Miller CJ (2003). Ethics: four levels for GP's. NZFP.

[B16] Morse Z, Makahara S (2001). English language education in Japanese dental schools. Eur J Dent Educ.

[B17] Maher J (1986). The development of English as an international language of medicine. Applied Linguistics.

[B18] Sehgal AR, Weisheit C, Miura Y, Butzlaff M, Kielstein R, Taguchi Y (1996). Advance directives and withdrawal of dialysis in the United States, Germany and Japan. JAMA.

[B19] Christakis NA (1992). Ethics are local: engaging cross cultural variation in the ethics for clinical research. Soc Sci Med.

[B20] Evans- Pritchard EE (1951). Social Anthropology.

[B21] Bradby H (2002). Translating culture and language:  A research note on multilingual settings. Sociology of Heath and Illness.

[B22] Boulet JR, van Zanten M, McKinley DW, Gary NE (2001). Evaluating the spoken English proficiency of graduates of foreign medical schools. Med Educ.

[B23] (1993). The manual for supervisors: institutional testing program.

[B24] SPSS for Windows (2001). Release 11.0.0 Standard Edition.

[B25] Goldie J, Schwartz L, McConnachie A, Morrison J (2002). The impact of three years' ethics teaching, in an integrated medical curriculum, on students' proposed behaviour on meeting ethical dilemmas. Med Educ.

[B26] Feldman MD, Zhang J, Cummings SR (1999). Chinese and U.S. internists adhere to different ethical standards. J Gen Intern Med.

[B27] Jackson T ( 2000). Management Ethics and Corporate Policy: A Cross-cultural Comparison. Journal of Management Studies.

[B28] Van Bommel A (1999). Medical ethics from the Muslim perspective. Acta Neurochir.

[B29] Aksoy S, Elmai A (2002). The core concepts of the 'four principles' of bioethics as found in Islamic tradition. Med Law.

[B30] Aksoy S,, Tenik A (2002). The 'four principles of bioethics' as found in 13th century Muslim scholar Mawlana's teachings. BioMed Central, Medical Ethics.

[B31] Daar AS,, Al Khitamy AB (2001). Bioethics for clinicians: 21. Islamic bioethics. Canadian Medical Association.

[B32] Ayres J.B, Peters R.M (1977). Predictive validity of the Test of English as a Foreign Language for Asian graduate students in engineering, chemistry, or mathematics. Educational and Psychological Measurement.

[B33] Al-Musawi NM, Al-Ansari SH (1999). Test of English as a Foreign Language and First Certificate of English tests as predictors of academic success for undergraduate students at the University of Bahrain. System.

[B34] Mpofu DJ, Lanphear J, Stewart T, Das M, Ridding P, Dunn E (1998). Facility with the English language and problem-based learning group interaction: findings from an Arabic setting. Med Educ.

